# One-Pot Deracemization of *sec*-Alcohols: Enantioconvergent Enzymatic Hydrolysis of Alkyl Sulfates Using Stereocomplementary Sulfatases**

**DOI:** 10.1002/ange.201209946

**Published:** 2013-02-10

**Authors:** Markus Schober, Michael Toesch, Tanja Knaus, Gernot A Strohmeier, Bert van Loo, Michael Fuchs, Florian Hollfelder, Peter Macheroux, Kurt Faber

**Affiliations:** M. Schober, M. Toesch, Dr. M. Fuchs, Prof. K. Faber Department of Chemistry, Organic & Bioorganic Chemistry, University of GrazHeinrichstrasse 28, 8010 Graz (Austria); Dr. T. Knaus, Prof. P. Macheroux Institute of Biochemistry, Graz University of Technology; Dr. G. A. Strohmeier ACIB GmbH c/o Department of Organic Chemistry, Graz University of Technology; Dr. B. van Loo, Prof. F. Hollfelder Department of Biochemistry, University of Cambridge

**Keywords:** Alkohole, Biotransformationen, Enzymkatalyse, Kinetische Racematspaltung, Synthesemethoden

Given the fact that the theoretically possible number of racemates is larger than that of symmetric prochiral or *meso* compounds,^1^ the development of deracemization methods, which yield a single stereoisomer from a racemate is an important topic.^1^–^3^ Enantioconvergent processes are based on the transformation of a pair of enantiomers through opposite stereochemical pathways affecting retention and inversion of configuration. Depending on the stereochemical course of enzymatic and chemical reactions, three types of deracemization protocols were recently classified by Feringa et al.^4^ Two chemoenzymatic methods start with a biocatalytic kinetic resolution step, which yields a hetero- or homochiral 1:1 mixture of the formed product and nonconverted substrate enantiomer. The latter is subjected to a second (non-enzymatic) transformation with retention or inversion of configuration to yield a single stereoisomeric product. Although several one-pot, two-step protocols have been successfully demonstrated,^5^, 10c,d they typically rely on activated species, such as sulfonates,5a–d nitrate esters,5b or Mitsunobu intermediates,5e and negatively affect the overall atom economy of the process. The most elegant method relies on one (or two) enzyme(s), which mediate the transformation of both enantiomers through stereocomplementary pathways by retention and inversion. Since the requirements of such double selectivities are very difficult to meet, successful examples are rare: This approach has been applied to the hydrolysis of epoxides using two epoxide hydrolases showing opposite enantiopreference^6^ or a single enzyme that catalyzes the enantioconvergent hydrolysis of enantiomers with opposite regioselectivity.^7^

For enzymes, the ability to act by retention or inversion is a rare feature, which has been found among epoxide hydrolases,^8^ dehalogenases,^4^, ^9^ and sulfatases.^10^ The latter catalyze the hydrolytic cleavage of (alkyl) sulfate esters by breakage of the S–O or the C–O bond leading to retention or inversion at the chiral carbon atom,10b and thus makes them prime candidates for enantioconvergent processes. So far, only a single inverting *sec*-alkylsulfatase (PISA1) was generated recombinantly and characterized biochemically,^11^ thus allowing preparative-scale applications.10c In combination with acid-catalyzed hydrolysis of the nonreacted substrate enantiomer under retention of configuration^12^ a chemoenzymatic two-step deracemization protocol for *sec*-alcohols was recently developed.10c,d However, the method suffers from serious limitations because it requires undesirably large volumes organic solvents and several molar equivalents of a strong acid (typically 2–7 equiv of *p*-TosOH), which pose the risk of racemization or decomposition to the functionalized substrates, especially when elevated temperatures are required for acidic hydrolysis. Moreover, it is not applicable to retaining sulfatases, because no chemical method for sulfate ester hydrolysis with inversion exists.10c

So far, retaining-sulfatase activity was reported in whole cells of *Rhodopirellula baltica* DSM 10527,^13^ but the corresponding enzymes could not be identified, thus impeding the use of recombinant technology to make the enzyme available for biocatalysis. Furthermore, the retaining sulfatase of *Rh. baltica* would not be suitable for an enantioconvergent process with PISA1, because both proteins exhibit the same enantiopreference. During our search for a retaining *sec*-alkylsulfatase with an enantiopreference opposite to that of PISA1, we discovered that the arylsulfatase from *Pseudomonas aeruginosa* (PAS) exhibited activity on *sec*-alkylsulfates. PAS, which has been characterized on a molecular level,^14^ showed promiscuous activity on various arylic phosphates and phosphonates.^15^ On its standard model substrate (4-nitrophenyl sulfate), PAS exhibited a rate acceleration of *k*_cat_/*k*_uncat_ 2.3×10^10^,^16^ and for a less reactive substrate the highest rate enhancement (*k*_cat_/*k*_uncat_=2×10^26^) of any catalytic reaction known so far has been measured.^17^ The stereochemical features of PAS were investigated using a series of *sec*-alkylsulfate esters (*rac*-**1 a**–**7 a**; Table 1). The substrates **1 a**–**3 a** bearing an acetylenic moiety on the long chain adjacent to the stereocenter were resolved with good to excellent enantioselectivities (*E* 59 to >200). Undesired non-enzymatic background hydrolysis of **1 a** could be suppressed by addition of 20 % (v/v) of DMSO as a cosolvent.^18^ In contrast, the selectivities were largely lost when the acetylenic moiety was moved to the short chain (substrates **4 a**–**6 a**). The alkyl aryl derivative **7 a** gave again an excellent *E* value of greater than 200. All substrates converted with high enantioselectivities (**1 a**–**3 a**, **7 a**) were hydrolyzed with complete retention of configuration, thus yielding *S*-configured alcohols and unreacted *R*-configured sulfate esters. To prove the stereochemical course of sulfate ester hydrolysis by PAS, enzymatic cleavage of *rac*-1-octyn-3-yl sulfate (**6 a**) was performed in an ^18^O-labeled buffer (label >98 %). GC/MS analysis of the alcohol **6 b** formed revealed that (in contrast to inverting *sec*-alkylsulfatases10c) no incorporation of ^18^O occurred, and is consistent with the attack of the enzyme’s formylglycine nucleophile on sulfur. Hydrolysis of enantiopure (*S*)-**6 a** by PAS yielded alcohol (*S*)-**6 b** in greater than 99 % *ee*, thus proving that hydrolysis proceeded under strict retention of configuration.

**Table 1:** Kinetic resolution of sulfate esters *rac*-1 a–7 a with retention and inversion using PAS and PISA1. 
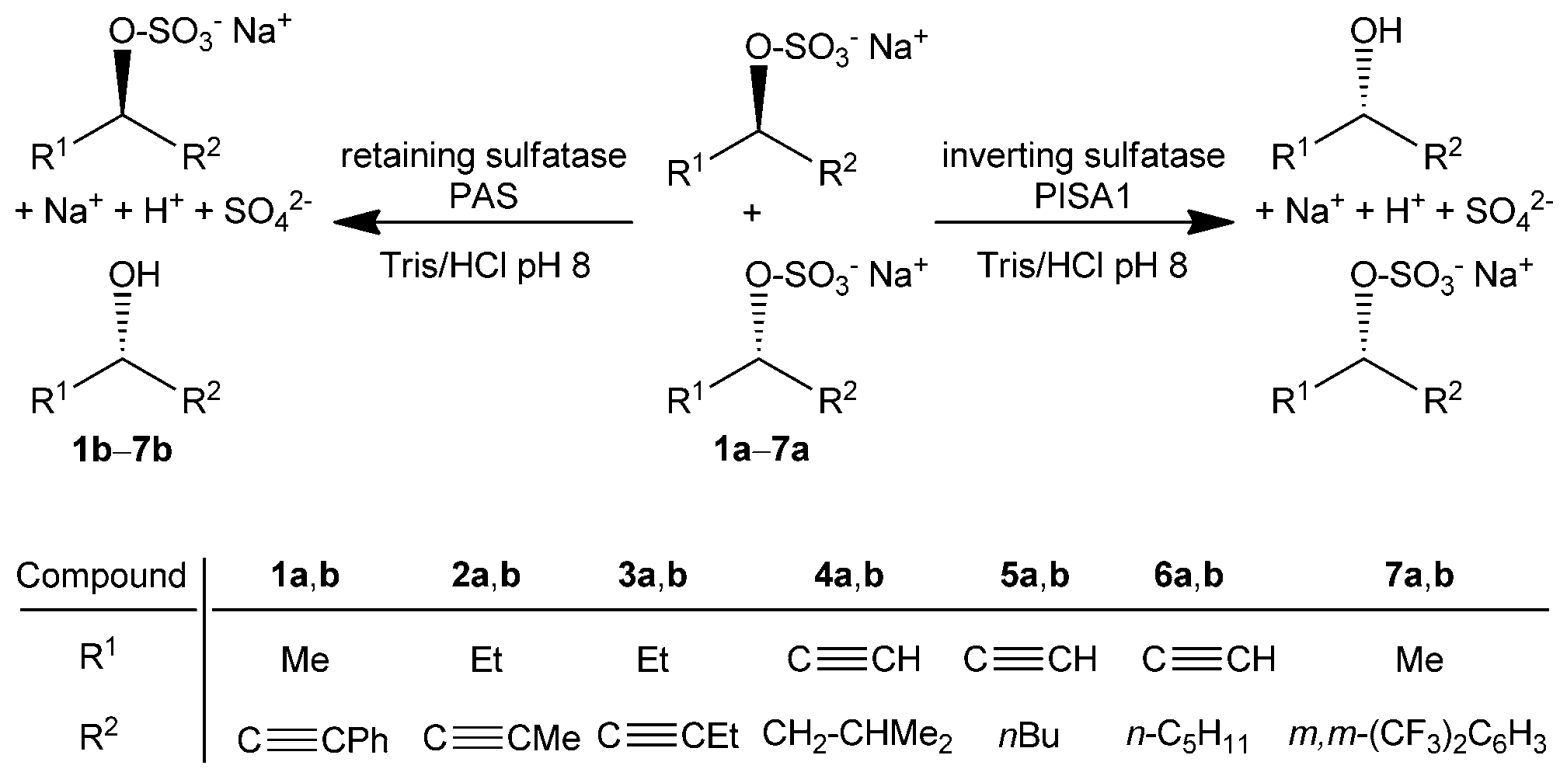

Substrate	Enzyme	*t* [h]	Conv. [%]^[a]^	*ee*_P_ [%]	*ee*_S_ [%]	*E* value^[a]^
*rac*-**1 a**^[b]^	PAS	24	49	97 (*S*)	92 (*R*)	190 (*S*)
*rac*-**2 a**	PAS	24	49	91 (*S*)	89 (*R*)	59 (*S*)
*rac*-**3 a**	PAS	6	46	98 (*S*)	84 (*R*)	>200 (*S*)
*rac*-**4 a**^[c^	PAS	6	61	48 (*S*)	74 (*R*)	6 (*S*)
*rac*-**5 a**^[c]^	PAS	6	53	11 (*S*)	11 (*R*)	<2 (*S*)
*rac*-**6 a**^[c]^	PAS	6	65	22 (*R*)	40 (*S*)	≍2 (*R*)
*rac*-**7 a**	PAS	48	30	>99 (*S*)	46 (*R*)	>200 (*S*)
*rac*-**1 a**^[b,d]^	PISA1	6	47	98 (*S*)	89 (*S*)	>200 (*S*)
*rac*-**2 a**^[d]^	PISA1	4	56	55 (S)	80 (*S*)	8 (*S*)
*rac*-**3 a**^[d]^	PISA1	6	57	32 (*S*)	40 (*S*)	≍3 (*S*)
*rac*-**4 a**^[c]^	PISA1	72	50	>99 (*R*)	>99 (*R*)	>200 (*R*)
*rac*-**5 a**^[c,e]^	PISA1	24	50	>99 (*R*)	>99 (*R*)	>200 (*R*)
*rac*-**6 a**^[c,d]^	PISA1	24	50	>99 (*R*)	>99 (*R*)	>200 (*R*)
*rac*-**7 a**	PISA1	72	49	>99 (*S*)	93 (*S*)	>200 (*S*)

See the Supporting Information for reaction conditions. [a] Calculated from *ee*_P_ (*ee* value of product) and *ee*_S_ (*ee* value of starting material) according to Ref. 10c,d. [b] 20 % DMSO (v/v) as cosolvent to suppress non-enzymatic background hydrolysis. [c] Switch in Cahn–Ingold–Prelog priorities. [d] See Ref. 10c. [e] See Ref. 10d.

Since the stereochemical features of PAS—*R* enantiopreference with retention—would ideally complement the *S* preference with inversion10c,d of PISA1,10e we optimized the enzymatic hydrolysis of the substrates **1 a**–**7 a** with the latter enzyme (Table 1). Most of the substrates (**1 a**, **4 a**–**7 a**) showed perfect *E* values of greater than 200, with only **2 a** and **3 a** giving insufficient selectivities. The data thus obtained enabled us to develop three types of enantioconvergent processes (Scheme 1, Table 2):

**Scheme 1:**
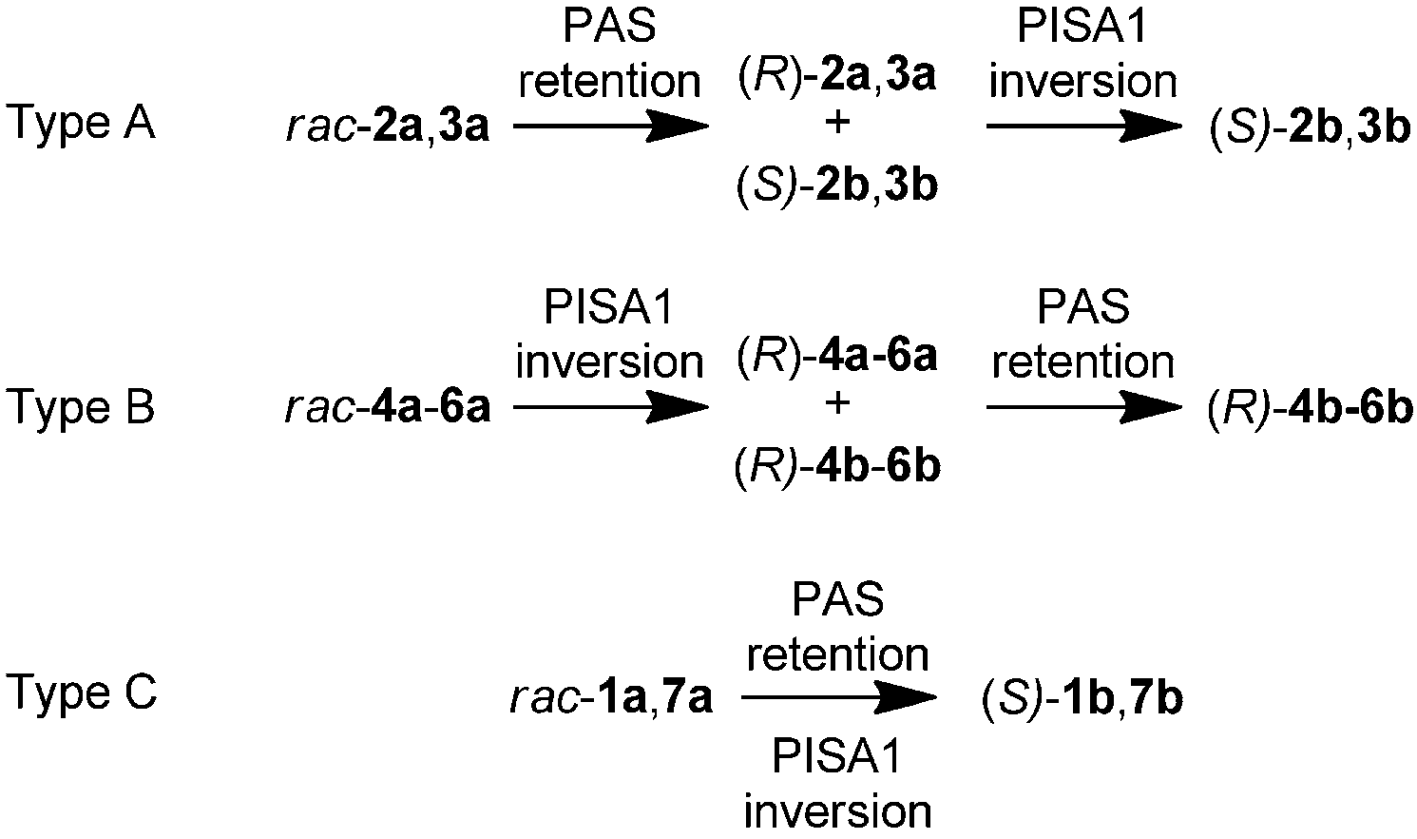
One-pot, two-enzyme deracemization process using retaining PAS and inverting PISA1.

**Table 2:** Deracemization of the sulfate esters *rac*-1 a–7 a using retaining PAS and inverting PISA1

Substrate	Reaction Type	*t*_PISA1_ [h]	*t*_PAS_ [h]	Conv. [%]	*ee*_P_ [%]
*rac*-**1 a**	C	24	24	93	93 (*S*)
*rac*-**1 a**^[a]^	C	24	24	98	95 (*S*)
*rac*-**1 a**^[a,b]^	C	24	24	93	98 (*S*)
*rac*-**2 a**	A	12	36	81	91 (*S*)
*rac*-**3 a**	A	6	18	>99	97 (*S*)
*rac*-**4 a**	B	72	24	>99	94 (*R*)
*rac*-**5 a**	B	48	24	>99	>99 (*R*)
*rac*-**6 a**	B	48	24	97	>99 (*R*)
*rac*-**7 a**	C	72	72	80	>99 (*S*)

See the Supporting Information for reaction conditions. [a] Double enzyme concentrations. [b] Cosolvent 20 % (v/v) DMSO.

- Type A. The substrates *rac*-**2 a** and *rac*-**3 a**, where PAS was highly enantioselective, could be deracemized by a one-pot, two-step sequence using retaining PAS first, followed by non-enantioselective inverting hydrolysis with PISA1 to yield (*S*)-**2 b** and (*S*)-**3 b** in 91 and 97 % *ee*, respectively.
- Type B. For *rac*-**4 a**–**6 a**, where PISA1 was highly enantioselective, but PAS was not, the opposite order of events—PISA1 first, PAS second—was successful and yielded the corresponding *R*-configured alcohols **4 b**–**6 b** in 94 to greater than 99 % *ee*
- Type C. The ideal single-step process using both enzymes simultaneously was designed for the substrates *rac*-**1 a** and *rac*-**7 a**. To maximize the *ee* value of **1 b**, DMSO was used as cosolvent to suppress background hydrolysis which increased the *ee* value of (*S*)-**1 b** from 93 to 98 % *ee*. To demonstrate the applicability of this method on a preparative scale, the deracemization of *rac*-**6 a** was scaled-up (1 g, 4.4 mmol), and gave (*R*)-**6 b** as the sole product in 82 % yield upon isolation with 98 % *ee*.

The choice of which process (Type A–C) is most suitable for the deracemization of a given substrate depends on the availability of an enantioselective *sec*-alkylsulfatase acting with retention or inversion of configuration. Processes of Types A and B are feasible with a single enantioselective enzyme, whereas Type C requires two enantioselective sulfatases with matching opposite enantiopreference. It should be kept in mind that Types A and B constitute kinetic resolutions,^19^ whereas Type C represents a parallel kinetic resolution.^20^

Overall, the purely enzymatic protocol excels by its significantly broader applicability compared to the chemoenzymatic procedure10c,d for the following reasons: 1) it eliminates the harsh reaction conditions required for acid-catalyzed hydrolysis, which are incompatible with sensitive functional groups and 2) it is also applicable to retaining sulfatases (such as PAS).

In summary, the one-pot deracemization of *sec*-alcohols bearing various functional groups was achieved by enantioconvergent hydrolysis of the corresponding sulfate esters using the retaining aryl sulfatase PAS and the inverting alkyl sulfatase PISA1, which possess the required opposite enantiopreference.

## Experimental Section

Preparative scale one-pot, one-step deracemization of *rac*-**6 a**: Purified PISA1 (0.5 mL, 13 mg, 176.5 nmol, 0.5 mL of stock solution) was added to 1-octyn-3-yl sulfate (*rac*-**6 a**, 1 g, 4.4 mmol) dissolved in Tris-HCl (197.5 mL, 100 mm, pH 8.0). The reaction was shaken at 120 rpm and 30 °C for 24 h. PAS (N-terminal strep tag, 2 mL, 26 mg, 434 nmol) was added. After an additional 24 h, the aqueous phase was extracted with *t*BuOMe (3×100 mL). The combined organic phases were dried with anhydrous Na_2_SO_4_ and filtered. The solvent was evaporated under reduced pressure (220 mbar, 30 °C) and (*R*)-**6 b** was obtained as a clear yellow oil (0.45 g, 3.6 mmol, 82 %) with the following physical properties: *ee* value 98 % [determined via GC as (*R*)-1-octyn-3-yl acetate]; 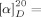
+4.6° (CHCl_3_, *c*=1.0); lit.^21^ 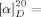
+5.3° (CHCl_3_, *c*=1.0); ^1^H NMR (300 MHz, CDCl_3_): *δ*=4.39 (dt, 29.0 and 9.2 Hz, 1 H), 2.48 (d, 5.2 Hz, 1 H), 1.80–1.67 (m, 2 H), 1.53–1.25 (m, 6 H), 0.92 ppm (t, 6.1 Hz, 3 H); ^13^C NMR (75 MHz, CDCl_3_): *δ*=85.0, 72.8, 62.3, 37.6, 31.4, 24.7, 22.5, 14.0 ppm.

## Supplementary material

As a service to our authors and readers, this journal provides supporting information supplied by the authors. Such materials are peer reviewed and may be re-organized for online delivery, but are not copy-edited or typeset. Technical support issues arising from supporting information (other than missing files) should be addressed to the authors.

## References

[1] Gruber CG, Lavandera I, Faber K, Kroutil W (2006). Adv. Synth. Catal.

[2a] Steinreiber J, Faber K, Griengl H (2008). Chem. Eur. J.

[2b] Turner NJ (2010). Curr. Opin. Chem. Biol.

[2c] Hein JE, Hyunh Cao B, Viedma C, Kellogg RM, Blackmond DG (2012). J. Am. Chem. Soc.

[2d] Carnell AJ (1999). Adv. Biochem. Eng./Biotechnol.

[4] Szymański W, Westerbeek A, Janssen DB, Feringa BL (2011). Angew. Chem.

[b01] (2011). Angew. Chem. Int. Ed.

[5a] Szymanski W, Ostaszewski R (2006). Tetrahedron: Asymmetry.

[5b] Danda H, Nagatomi T, Maehara A, Umemura T (1991). Tetrahedron.

[5c] Lemke K, Ballschuh S, Kunath A, Theil F (1997). Tetrahedron: Asymmetry.

[5d] Vänttinen E, Kanerva LT (1995). Tetrahedron: Asymmetry.

[5e] Takano S, Suzuki M, Ogasawara K (1993). Tetrahedron: Asymmetry.

[5f] Orru RVA, Mayer SF, Kroutil W, Faber K (1998). Tetrahedron.

[6a] Pedragosa-Moreau S, Archelas A, Furstoss R (1993). J. Org. Chem.

[6b] Cao L, Lee JT, Chen W, Wood TK (2006). Biotechnol. Bioeng.

[7a] Kroutil W, Mischitz M, Faber K (1997). J. Chem. Soc. Perkin Trans. 1.

[7b] Monterde MI, Lombard M, Archelas A, Cronin A, Arand M, Furstoss R (2004). Tetrahedron: Asymmetry.

[7c] Hwang S, Choi CY, Lee EY (2008). Biotechnol. Lett.

[8a] Steinreiber A, Faber K (2001). Curr. Opin. Biotechnol.

[8b] Bala N, Chimni SS (2010). Tetrahedron: Asymmetry.

[8c] de Vries EJ, Janssen DB (2003). Curr. Opin. Biotechnol.

[9a] Koudelakova T, Bidmanova S, Dvorak P, Pavelka A, Chaloupkova R, Prokop Z, Damborsky J (2013). Biotechnol. J.

[9b] Soda K, Kurihara T, Liu J-Q, Nardi-Dei V, Park C, Miyagi M, Tsunasawara S, Esaki N (1996). Pure Appl. Chem.

[10a] Pogorevc M, Kroutil W, Wallner SR, Faber K (2002). Angew. Chem.

[b02] (2002). Angew. Chem. Int. Ed.

[10b] Gadler P, Faber K (2007). Trends Biotechnol.

[10c] Schober M, Gadler P, Knaus T, Kayer H, Birner-Gruenberger R, Guelly C, Macheroux P, Wagner U, Faber K (2011). Org. Lett.

[10d] Schober M, Knaus T, Toesch M, Macheroux P, Wagner U, Faber K (2012). Adv. Synth. Catal.

[11] Knaus T, Schober M, Kepplinger B, Faccinelli M, Pitzer J, Faber K, Macheroux P, Wagner U (2012). FEBS J.

[12] Wallner SR, Nestl B, Faber K (2005). Tetrahedron.

[13] Wallner SR, Batter M, Würdemann C, Wecker P, Glöckner FO, Faber K (2005). Angew. Chem.

[b03] (2005). Angew. Chem. Int. Ed.

[14] Boltes I, Czapinska H, Kahnert A, von Bülow R, Dierks T, Schmidt B, von Figura K, Kertesz MA, Uson I (2001). Structure.

[15a] Babtie AC, Bandyopadhyay S, Olguin LF, Hollfelder F (2009). Angew. Chem.

[b04] (2009). Angew. Chem. Int. Ed.

[15b] Olguin LF, Askew SE, O’Donoghue AC, Hollfelder F (2008). J. Am. Chem. Soc.

[15c] Kintses B, Hein C, Mohamed MF, Fischlechner M, Courtois F, Laine C, Hollfelder F (2012). Chem. Biol.

[16] Jonas S, Hollfelder F (2009). Pure Appl. Chem.

[17] Edwards DR, Lohman DC, Wolfenden R (2012). J. Am. Chem. Soc.

[19] Pedragosa-Moreau S, Morisseau C, Baratti J, Zylber J, Archelas A, Furstoss R (1997). Tetrahedron.

[20] Eames J (2000). Angew. Chem.

[b05] (2000). Angew. Chem. Int. Ed.

[21] Bischop M, Doum V, Rieche ACMNordschildnée, Pietruszka J, Sandkuhl D (2010). Synthesis.

