# Supplementary material for: One-Pot Deracemization of sec-Alcohols: Enantioconvergent Enzymatic Hydrolysis of Alkyl Sulfates Using Stereocomplementary Sulfatases
Source: Angew Chem Weinheim Bergstr Ger. 2013 Feb 10;125(11):3359–61. doi: 10.1002/ange.201209946 (PMC4373141; doi:10.1002/ange.201209946)
Supplement: Supplementary file 1 [file ange0125-3359-SD1.pdf]

Angewandte Chemie
Eine Zeitschrift der Gesellschaft Deutscher Chemiker

Supporting Information
© Wiley-VCH 2013
69451 Weinheim, Germany

# One-Pot Deracemization of *sec*-Alcohols: Enantioconvergent Enzymatic Hydrolysis of Alkyl Sulfates Using Stereocomplementary Sulfatases**

*Markus Schober, Michael Toesch, Tanja Knaus, Gernot A. Strohmeier, Bert van Loo, Michael Fuchs, Florian Hollfelder, Peter Macheroux, and Kurt Faber**

ange_201209946_sm_miscellaneous_information.pdf

## Table of Contents

General .......... 2
Synthesis of alkyl sulfate esters .......... 2
Cloning, expression and purification of PISA1 .......... 3
Expression and purification of *Pseudomonas aeruginosa* arylsulfatase (PAS) .......... 3
Determination of protein concentrations .......... 3
Enantioselectivity assays for PISA1 .......... 4
Enantioselectivity assays for PAS .......... 4
Determination of absolute configuration .......... 4
Determination of enantiomeric excess of products .......... 4
Enantioselectivity assays for the enantioconvergent process .......... 5
  Determination of conversion .......... 5
  Determination of enantiomeric excess .......... 6
$^{18}OH_2$ Labeling experiments .......... 6
NMR spectra of preparative biotransformations .......... 7
MS-spectra of $^{18}O$ labeling studies .......... 8
References .......... 11

## General

Competent cells One Shot® TOP10 and One Shot® BL21 Star™ (DE3) from Invitrogen were transformed according to the manufacturer´s protocol. Secondary alcohols **1b**-**7b** were purchased from Sigma Aldrich, Acros and Alfa Aesar. $^{18}OH_2$ for the preparation of $^{18}O$-labeled buffer was from Rotem (>98%). NMR spectra were recorded on a Bruker spectrometer at 300 ($^1H$) and 75 ($^{13}C$) MHz. Chemical shifts (δ) are given in ppm and coupling constants (*J*) are given in Hz. Optical rotation values were determined on a Perkin Elmer Polarimter 341. GC-MS measurements were performed on an Agilent 7890A GC system, equipped with an Agilent 5975C mass-selective detector (EI 70 eV) and a HP-5-MS column (30 m x 0.25 mm x 0.25 μm film) using helium gas at a flow rate of 0.55 mL/min. GC-measurements were conducted using an Agilent Technologies 7890A GC-FID system equipped with an Agilent Technologies 7683B autosampler and a Varian Chirasil Dex CB column (25 m x 0.32 mm x 0.25 μm film). HPLC-MS measurements were done on a Shimadzu Nexera instrument equipped with a Shimadzu LCMS-2020 MS-detector and a Machery Nagel EC 150/4 Nucleosil® 120-5 C4 column (150 mm x 4 mm). Further details are shown below.

## Synthesis of alkyl sulfate esters

Sulfate esters *rac*-**1a**-**7a**, (*R*)-and (S*)*-**6a** were prepared from the corresponding alcohols by using $NEt_3*SO_3$ following a known procedure.[1,2]

### NMR Data for alkyl sulfates

*rac*-4-Phenyl-3-butyn-2-yl sulfate (**1a**)

$^1H$ NMR (300 MHz, $D_2O$): d = 7.46-7.22 (m, 5H), 5.19 (q, *J* = 38.8 Hz, 1H), 1.51 (d, *J* = 10.0 Hz, 3H); $^{13}C$ NMR (75 MHz, $D_2O$): d =131.6, 129.1, 128.5, 121.3, 87.2, 85.8, 66.4, 21.7.

*rac*-4-Hexyn-3-yl sulfate (**2a**)

$^1H$ NMR (300 MHz, $D_2O$): d = 4.85-4.77 (m, 1H), 1.82-1.68 (m, 5H), 0.92 (t, *J* = 6.9 Hz, 3H); $^{13}C$ NMR (75 MHz, $D_2O$): d = 84.5, 76.6, 71.5, 28.9, 8.5, 2.6.

*rac*-4-Heptyn-3-yl sulfate (**3a**)

$^1H$ NMR (300 MHz, $D_2O$): d = 4.88-4.80 (m, 1H), 2.25-2.14 (m, 2H), 1.79-1.72 (m, 2H), 1.06 (t, *J* = 8.0 Hz, 3H), 0.94 (t, *J* = 7.2 Hz, 3H); $^{13}C$ NMR (75 MHz, $D_2O$): d = 90.2, 76.9, 71.4, 29.0, 12.9, 11.7, 8.6.

*rac*-5-Methyl-1-hexyn-3-yl sulfate (**4a**)

$^1H$ NMR (300 MHz, $D_2O$): d = 4.68 (dt, *J* = 33.7 and 9.8 Hz, 1H), 3.27 (d, 6.9 Hz, 1H), 1.84-1.55 (m, 2H), 1.49-1.39 (m, 1H), 0.92-0.82 (m, 6H); $^{13}C$ NMR (75 MHz, $D_2O$): d =84.4, 75.5, 64.8, 45.1, 24.5, 23.1, 22.6.

*rac*-1-Heptyn-3-yl sulfate (**5a**)

$^1H$ NMR (300 MHz, DMSO-$d_6$): δ = 4.67-4.62 (m, 1H), 3.28 (d, 7.9 Hz, 1H), 1.70-1.53 (m, 2H), 1.42-1.21 (m, 4H), 0.86 (t, 5.9 Hz, 3H); $^{13}C$ NMR (75 MHz, DMSO-$d_6$): δ = 84.2, 75.6, 66.0, 35.7, 27.0, 22.3, 14.4.

*rac*-1-Octyn-3-yl sulfate (**6a**)

$^1$H NMR (300 MHz, DMSO-$d_6$): δ = 4.67-4.62 (m, 1H), 3.24 (d, 6.8 Hz, 1H), 1.68-1.51 (m, 2H), 1.42-1.16 (m, 6H), 0.83 (t, 5.6 Hz, 3H); $^{13}$C NMR (75 MHz, DMSO-$d_6$): δ = 83.8, 75.7, 66.4, 35.8, 31.2, 24.3, 22.4, 14.3.

*rac*-1-[3,5-Bis(trifluoromethyl)phenyl]ethyl sulfate (**7a**)

$^1$H NMR (300 MHz, DMSO-$d_6$): d = 8.02 (s, 2H), 7.96 (s, 1H), 5.45-5.37 (q, 35.7 Hz, 1H), 1.46 (d, 9.6 Hz, 3H); $^{13}$C NMR (75 MHz, DMSO-$d_6$): d =148.3, 131.0, 130.6, 130.1, 129.7, 127.1, 125.7, 122.1, 72.6, 23.9.

**Cloning, expression and purification of PISA1**

PISA1 was obtained according to previously published procedures.[1,2]

**Expression and purification of *Pseudomonas aeruginosa* arylsulfatase (PAS)**

*E. coli* BL21 (DE3) was transformed with PAS harboring a N-terminal strep-tag (pASK-IBA5plus vector with *Eco*RI and *Hind*III as the respective restriction sites). Cells were grown in LB-medium containing ampicillin (100 µg/mL) at 37 °C and 120 rpm until the culture reached an $A_{600}$ of 0.6. After cooling to 30 °C, expression was induced with anhydrotetracyclin (200 µg/L). After 6 h, cells were harvested at 4 °C and 8000 rpm for 10 min. The cell pellet was washed with NaCl (0.9%) and stored at -20° C. After resuspension in Tris-HCl buffer (100 mM, 150 mM NaCl, pH 8.0), cells were disrupted by sonication using a Sonics & Materials Vibra Cell CV26 (13 mm tip, 5 min, 40% amplitude, pulse 1 sec on, 2 sec off). After centrifugation (4 °C, 18000 rpm, 2x15 min), the supernatant was filtered through a MN 615 filter paper (Machery Nagel) and subjected to strep-tag purification on a 5 mL gravity flow column according to the protocol of the supplier (IBA BioTAGnology). The protein was eluted with 6 x 2.5 mL of EDTA-free elution buffer (100 mM Tris-HCl pH 8.0, 150 mM NaCl, 2.5 mM desthiobiotin). The combined elution fractions were afterwards dialyzed in 5 L of Tris-HCl (100 mM, pH 8.0) overnight and concentrated with a Vivaspin centrifugal concentrator (10,000 MWCO) to a final concentration of ~20 mg/mL. Aliquots of the concentrated enzyme solution were shock frozen in liquid nitrogen and stored at -20 °C. The yield was ca. 100 mg/L culture.

**Determination of protein concentrations**

Protein concentrations were determined by measuring the absorbance at 280 nm on an Eppendorf Biophotometer plus spectrophotometer. Purified protein solution (60 µL, diluted to fit into the linear range of the spectrophotometer) was directly measured in an UV permeable cuvette with buffer from the preceding dialysis used for blank determination and dilution of the sample. The concentration was calculated according to Lambert-Beer's law using an extinction coefficient obtained from the ProtParam tool from ExPASy ($\varepsilon^{280}$= 102790 $M^{-1}$ $cm^{-1}$).[3]

**Enantioselectivity assays for PISA1**

Enantioselectivity assays were conducted according to a known procedure[1] with slight modifications: 20 mg of substrate was used and quantities for all other reactants were adapted accordingly. Reaction were conducted with 1.77 nM PISA1 for substrates *rac*-**1a**, -**2a**, -**3a**, -**5a** and -**6a**, and with 7.08 nM PISA1 for *rac*-**4a** and -**7a**. Reaction times, enantiomeric excesses and *E*-values are found in the main paper.

**Enantioselectivity assays for PAS**

Enantioselectivity assays have been conducted as described above for PISA1.[1] Reactions were conducted with 3.54 nM PISA1 for substrates *rac*-**1a**-**7a**. Reaction times, enantiomeric excess and *E*-values are displayed in the main text.

**Determination of absolute configuration**

Absolute configurations of products **1b**-**7b** were determined after derivatization to the corresponding acetates **1c**-**7c** as previously shown.[1,2]

**Determination of enantiomeric excess of products**

The enantiomeric excess of alcohols **1b–7b** was determined *via* GC after derivatization to the corresponding acetates **1c**-**7c** using an Agilent Technologies 7890A GC-FID system equipped with an Agilent Technologies 7683B autosampler and a Varian Chirasil Dex CB column (25 m x 0.32 mm x 0.25 μm film) as previously shown.[1] The following methods were used:

| Method | Injector temperature [°C] | He Flow rate [mL/min] | Temperature programme | | | |
|---|---|---|---|---|---|---|
| | | | Step 1 | Step 2 | Step 3 | Step4 |
| A | 200 | 2.0 | 80 °C, hold 1.0 min | 15 °C/min to 110 °C | 4 °C/min to 130 °C | 10 °C/min to 180 °C |
| B | | | | 3 °C/min to 100 °C | 15 °C/min to 150 °C | |
| C | | | | 15 °C/min to 140 °C | 4 °C/min to 160 °C | 10 °C/min to 180 °C |
| D | | | | 3 °C/min to 95 °C | 15 °C/min to 150 °C | |

**Table 1:** GC-Data of acetates **1c**-**7c**.

| Compound | Method | Retention Time [min] | |
|---|---|---|---|
| 4-Phenyl-3-butyn-2-yl acetate (**1c**) | C | 8.3 (*S*) | 8.4 (*R*) |
| 4-Hexyn-3-yl acetate (**2c**) | D | 4.9 (*S*) | 5.2 (*R*) |
| 4-Heptyn-3-yl acetate (**3c**) | B | 5.9 (*S*) | 6.4 (*R*) |
| 5-Methyl-1-hexyn-3-yl acetate (**4c**) | B | 5.0 (*R*)* | 5.5 (*S*)* |
| 1-Heptyn-3-yl acetate (**5c**) | B | 6.3 (*R*)* | 7.3 (*S*)* |
| 1-Octyn-3-yl acetate (**6c**) | A | 5.5 (*R*)* | 5.9 (*S*)* |
| 1-[3,5-Bis(trifluoromethyl) phenyl]ethyl acetate (**7c**) | A | 4.5 (*S*) | 4.8 (*R*) |

* Switch in Cahn-Ingold-Prelog priority.

**Enantioselectivity assays for the enantioconvergent process**

Alkyl sulfate *rac*-**1a**-**7a** (5 mg) was dissolved in Tris-HCl (100 mM, pH 8.0) and an aliquot of PISA1 and PAS enzyme solution (**Table 2**) was added to a total volume of 1 mL. The reaction mixture was shaken at 30 °C and 120 rpm for the respective reaction times (Table 2 main paper).

***Determination of conversion***

Tris-HCl buffer (200 µL, 100 mM, pH 8.0), formic acid (200 µL from a 5 M stock in 100 mM Tris-HCl, pH 8.0) and internal standard (100 µL from a 10 mg/mL stock solution of sodium dodecyl sulfate) were added to 500 µL of the reaction. 10 µL of this solution were diluted with double distilled $H_2O$ (990 µL), 500 µL of the diluted sample were centrifuged at 4 °C and 13000 rpm for 15 min in a centrifugal filter (VWR, 3 kDa MW cutoff) to remove residual protein. The residual substrate concentration was determined by quantitative analysis on a Shimadzu Nexera instrument equipped with a Shimadzu LCMS-2020 MS-detector and a Machery Nagel EC 150/4 Nucleosil® 120-5 C4 column (150 mm x 4 mm). Conversions were calculated from the respective Selected Ion Monitoring (SIM) signals using sodium dodecyl sulfate (SDS, 1 mg/mL) as internal standard. Calibration curves were obtained from data points at 0.1, 0.5, 1.0, 2.0 and 5.0 mg/mL substrate. Retention times of substrates and the internal standard can be found in **Table 2**. The following methods were used:

*General HPLC-set-up:* flow rate: 1.0 mL/min, column oven temperature 40 °C; solvent A: aqueous 0.01% HCOOH, solvent B: acetonitrile; MS: negative ion mode, SIM measurement every 0.1 sec, detector voltage 0.9 kV. Gradient: 30% B for 4.0 min, gradient to 100% B for 2 min, 100% B for 1 min, 5% B for 3 min. Injection volume: 1 µL.

LC/Mass spectrometic characterization of substrates **1a**-**7a**.

| Substrate | SIM signals (Da): |
|---|---|
| **1a** | 224, 225 (substrate), 226, 248, 264, 265 (SDS), 266 |
| **2a** | 176, 177 (substrate), 178, 200, 264, 265 (SDS), 266. |
| **3a-5a** | 190, 191 (substrate), 192, 214, 264, 265 (SDS), 266. |
| **6a** | 204, 205 (substrate), 206, 228, 264, 265 (SDS), 266 |
| **7a** | 336, 337 (substrate), 338, 360, 264, 265 (SDS), 266. |

**Table 2:** Reaction conditions of the one-pot enantio-convergent processes using PISA1 and PAS.

| Substrate | $t_S$ [min] | $t_{IS}$ [min] | Conc. PISA1 [nM] | Conc. PAS [nM] |
|---|---|---|---|---|
| *rac*-**1a** | | | 1.77 | 3.54 |
| *rac*-**1a**[a] | 1.7 | 6.0 | 3.54 | 7.08 |
| *rac*-**1a** | | | 3.54 | 7.08 |
| *rac*-**2a** | 1.7 | 5.9 | 1.77 | 3.54 |
| *rac*-**3a** | 1.8 | 5.9 | 1.77 | 3.54 |
| *rac*-**4a** | 1.7 | 5.9 | 7.08 | 3.54 |
| *rac*-**5a** | 1.8 | 5.9 | 1.77 | 3.54 |
| *rac*-**6a** | 1.7 | 6.0 | 1.77 | 3.54 |
| *rac*-**7a** | 6.3 | 6.4 | 7.08 | 3.54 |

[a] Cosolvent DMSO 20% (v/v).

***Determination of enantiomeric excess***

The residual 500 µL of the reaction mixture were extracted with ethyl acetate (750 µL) and the organic phase was dried over anhydrous sodium sulfate. The enantiomeric excess of alcohols **1b**-**7b** was determined after derivatization to the corresponding acetates **1c**-**7c** as shown above.

## $^{18}OH_2$ Labeling experiments

Enzymatic assays of alkyl sulfate *rac*-**6a** (2.5 mg, 11 µmol) were conducted in unlabeled and $^{18}$O-labeled Tris-HCl (500 µL, 100 mM, pH 8.0) with PAS and PISA1. The reaction mixture was shaken at 30 °C and 120 rpm for 24 h. After extraction with ethyl acetate (500 µL), the organic phase was dried over anhydrous sodium sulfate. Product **6b** was derivatized to the corresponding acetate **6c** and analyzed on an Agilent 7890A system equipped with an achiral HP-5 column (30 m x 0.25 mm x 0.25 µm film) and an Agilent 5975C mass selective detector (electron impact, 70 eV) using He as carrier gas. The retention time for **6c** was 4.8 min. The following method was used: injector temperature 250 °C, flow 0.5 mL/min; temperature program: 100 °C, hold for 0.5 min, 10 °C/min, to 300 °C.

## NMR spectra of preparative biotransformations

$^{1}$H-NMR of (*R*)-**6b**

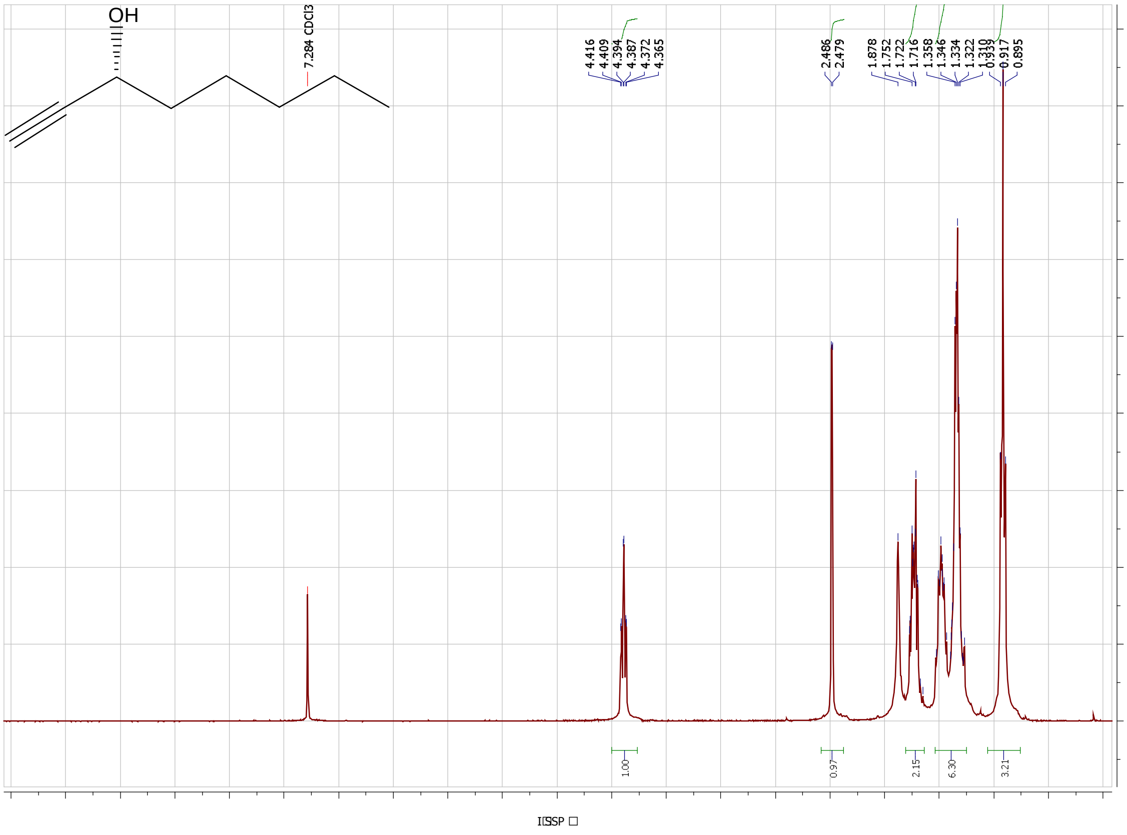


$^{13}$C-NMR of (*R*)-**6b**

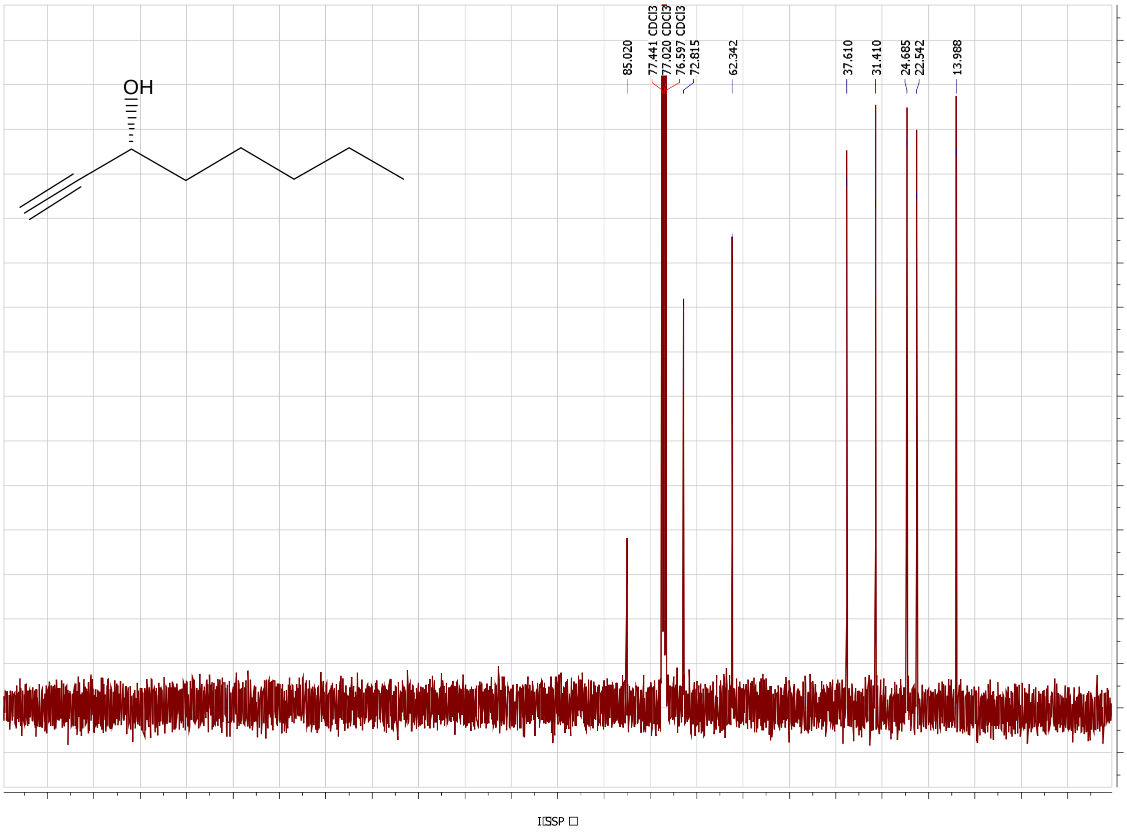

## MS-spectra of $^{18}$O labeling studies

MS-spectrum of **6c** from biotransformations with PAS in unlabeled buffer

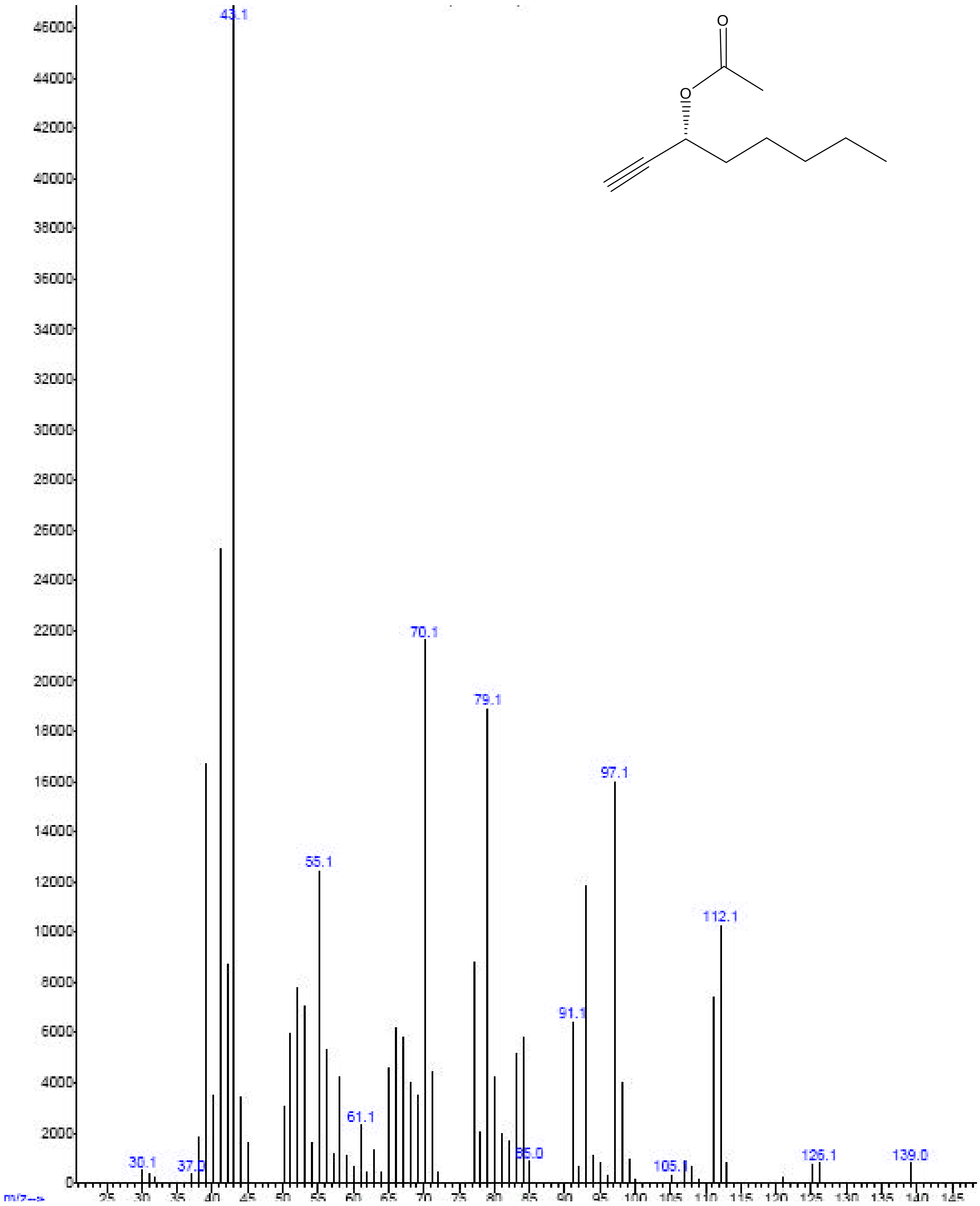

MS-spectrum of **6c** from biotransformations with PAS in $^{18}$O-labeled buffer

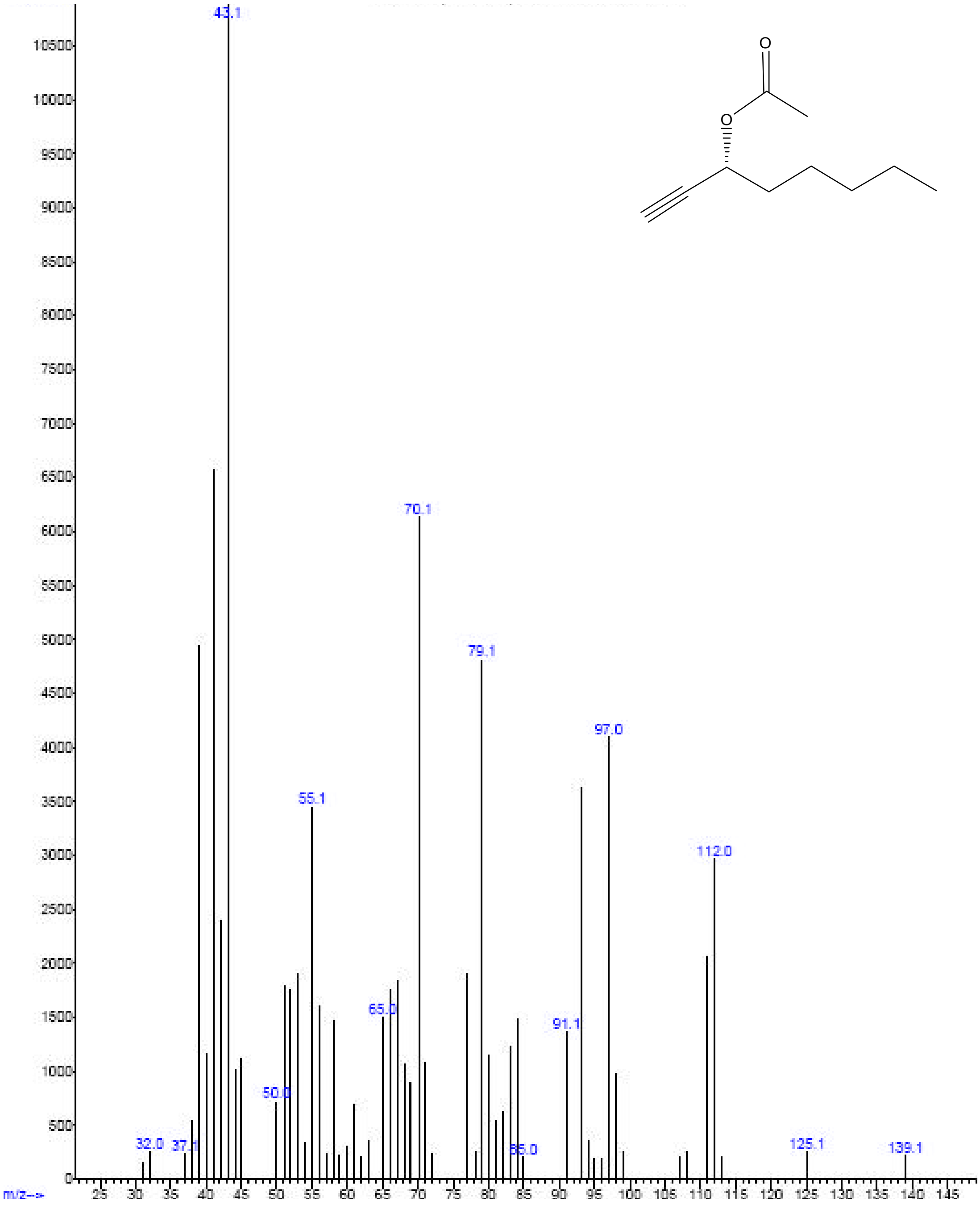

MS-spectrum of **6c** from biotransformations with PISA1 in unlabeled buffer

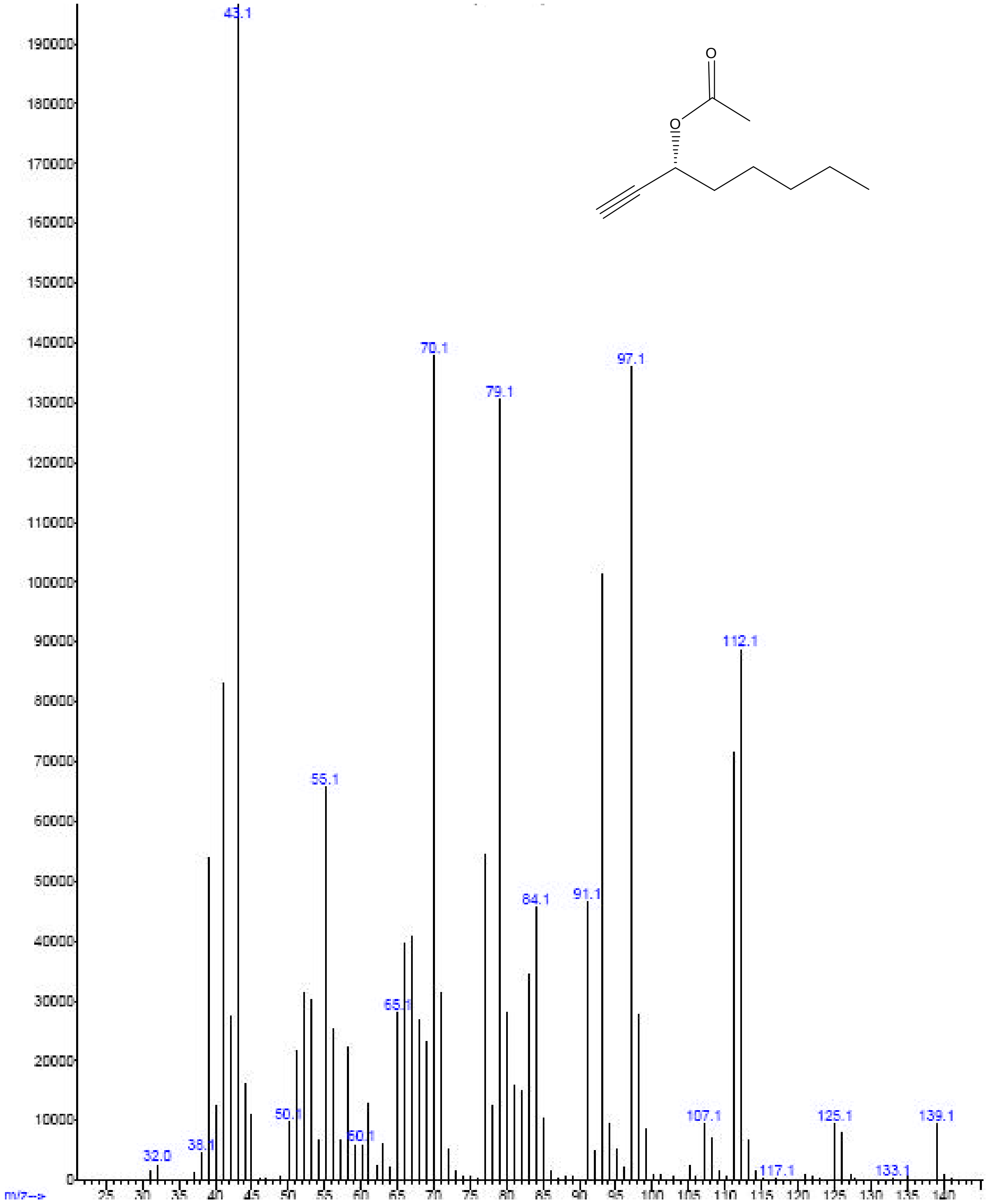

MS-spectrum of **6c** from biotransformations with PISA1 in $^{18}$O-labeled buffer

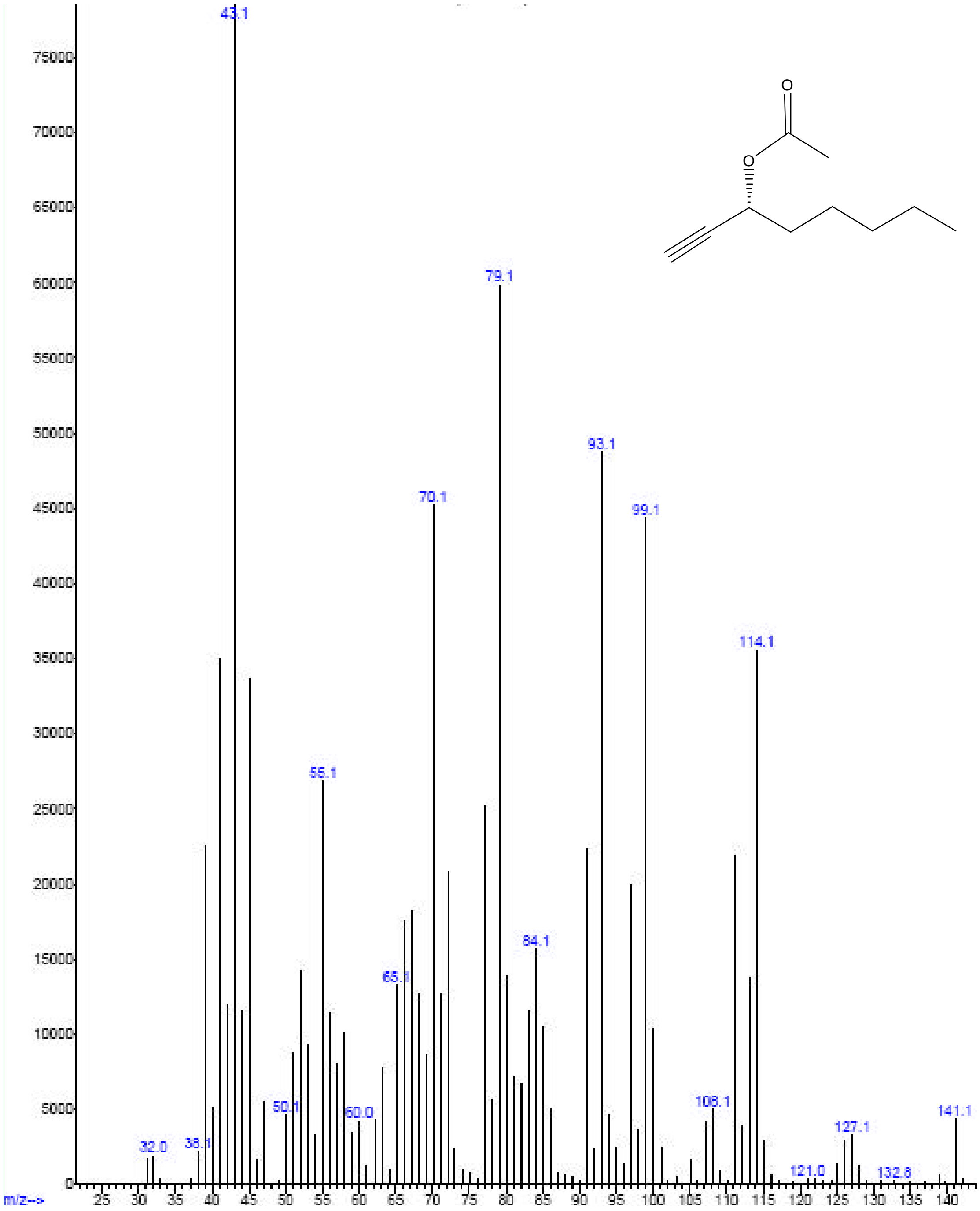


**References**


[1] M. Schober, P. Gadler, T. Knaus, H. Kayer, R. Birner-Gruenberger, C. Guelly, P. Macheroux, U. Wagner, K. Faber, *Org. Lett.* **2011**, *13*, 4296-4299.

[2] M. Schober, T. Knaus, M. Toesch, P. Macheroux, U. Wagner, K. Faber, *Adv. Synth. Catal.* **2012**, *354*, 1737-1742.

[3] http://web.expasy.org/protparam/.
